# Takotsubo Syndrome Mimicking Acute Myocardial Infarction: A Case Report

**DOI:** 10.7759/cureus.105641

**Published:** 2026-03-22

**Authors:** Jesus Miguel Figueroa Zaldivar, Marco Antonio Rodríguez Sánchez, Victor Emmanuel Gonzalez Zazueta, David Alejandro González Carrillo, José Manuel Aguilar Rubio, Yolanda Mercado Heredia, Carlos Orlando López Millán

**Affiliations:** 1 Department of Internal Medicine, Center for Research and Teaching in Health Sciences, Autonomous University of Sinaloa, Civil Hospital of Culiacán, Culiacán, MEX; 2 Department of Cardiology, Center for Research and Teaching in Health Sciences, Autonomous University of Sinaloa, Civil Hospital of Culiacán, Culiacán, MEX

**Keywords:** acute coronary syndrome (acs), acute myocardial infarction mimic, apical ballooning, minoca, stress-induced cardiomyopathy, takotsubo syndrome

## Abstract

Takotsubo syndrome is an acute, stress-related cardiomyopathy characterized by transient left ventricular systolic dysfunction in the absence of obstructive coronary artery disease. Although often clinically indistinguishable from acute coronary syndrome, Takotsubo syndrome represents a distinct form of myocardial injury within the Myocardial Infarction with Non-Obstructive Coronary Arteries (MINOCA) spectrum. Prompt recognition is essential, as early complications may include hemodynamic instability and clinically significant arrhythmias.

We present the case of a 77-year-old woman with type 2 diabetes mellitus, systemic arterial hypertension, and a prior ischemic stroke who was admitted with intense retrosternal chest pain of a six-hour duration. Laboratory testing demonstrated elevated high-sensitivity cardiac troponin and markedly increased pro-B-type natriuretic peptide levels. Electrocardiography revealed ischemic repolarization abnormalities. Transthoracic echocardiography identified segmental systolic dysfunction involving the mid-to-apical segments with preserved basal contractility. Invasive coronary angiography excluded significant epicardial coronary stenosis, while left ventriculography demonstrated apical ballooning with basal hypercontractility, confirming the diagnosis of Takotsubo syndrome.

This case highlights the importance of integrating clinical findings, biomarker profiles, and multimodality imaging in elderly women presenting with suspected acute coronary syndrome and non-obstructive coronary arteries.

## Introduction

Takotsubo syndrome (TTS), also referred to as stress-induced cardiomyopathy or “broken heart syndrome,” is an acute and typically reversible disorder characterized by transient systolic dysfunction of the left ventricle in the absence of flow-limiting coronary artery disease. The condition is defined by regional wall motion abnormalities that extend beyond the distribution of a single epicardial coronary artery, most frequently producing the classic pattern of apical ballooning. TTS is estimated to account for approximately 1-2% of patients evaluated for suspected acute coronary syndrome and occurs predominantly in postmenopausal women [1].

The underlying mechanisms are complex and not yet fully clarified. Current evidence supports a central role of sympathetic overactivation and catecholamine excess triggered by emotional or physical stress, resulting in myocardial stunning, microvascular impairment, and metabolic disturbance at the cellular level [2]. Clinically, patients often present with acute chest pain accompanied by electrocardiographic repolarization abnormalities and elevation of cardiac biomarkers, creating a presentation that is frequently indistinguishable from myocardial infarction [2]. Diagnosis relies on the integration of clinical findings with imaging evidence of transient ventricular dysfunction and the exclusion of obstructive coronary disease on angiography, followed by documented recovery of systolic function.

Although the overall prognosis is favorable in most cases, the acute phase may be complicated by heart failure, malignant arrhythmias, or hemodynamic instability, underscoring the importance of early identification and appropriate supportive management [3]. Given its close resemblance to acute myocardial infarction and the diagnostic challenges it poses, the presentation of illustrative cases remains valuable, particularly in populations that are underrepresented in existing reports.

## Case presentation

We describe a 77-year-old woman with a medical history significant for type 2 diabetes mellitus, long-standing systemic arterial hypertension, major depressive disorder, and a prior ischemic cerebrovascular event. Her chronic medications included fluoxetine, insulin glargine, losartan, and nifedipine. She was also receiving acetylsalicylic acid and atorvastatin for secondary stroke prevention. Diabetic peripheral neuropathy had been previously diagnosed and was managed with gabapentin.

She sought emergency care due to abrupt-onset, intense retrosternal chest pain rated 10/10 in severity, which had started approximately six hours before her arrival at the hospital. The pain radiated to the dorsal region and was accompanied by nausea without emesis. At presentation, her symptoms raised a strong suspicion of an acute coronary event. She was admitted to the emergency department with the following vital signs: heart rate of 85 beats per minute (reference range: 60-100 beats per minute), blood pressure of 133/91 mmHg (reference value: <120/80 mmHg), respiratory rate of 18 breaths per minute (reference range: 12-20 breaths per minute), peripheral oxygen saturation of 96% (reference value: ≥95%), and a temperature of 36°C (reference range: 36.0-37.5 °C).

Initial laboratory evaluation showed mild normocytic, normochromic anemia (hemoglobin 10.8 g/dL) with preserved renal function (urea 35 mg/dL, creatinine 0.7 mg/dL). Cardiac biomarkers demonstrated a significant rise in high-sensitivity troponin (4,800 ng/L; 99th percentile upper reference limit <34 ng/L), with a dynamic rising and falling pattern consistent with acute myocardial injury. Additionally, n-terminal pro B-type natriuretic peptide (NT-proBNP) was markedly elevated at 8,448 pg/mL. These findings supported the presence of acute myocardial injury (Table 1).

**Table 1:** Initial laboratory findings on admission Reference ranges may vary depending on the laboratory and assay used.

Laboratory parameter	Result	Reference range
Hemoglobin	10.8 g/dL	12.0–16.0 g/dL
Urea	35 mg/dL	15–45 mg/dL
Creatinine	0.7 mg/dL	0.6–1.2 mg/dL
High-sensitivity cardiac troponin	4,800 ng/L	<99th percentile (assay-specific)
Pro–B-type natriuretic peptide (pro-BNP)	8,448 pg/mL	<300 pg/mL

**Figure 1:**
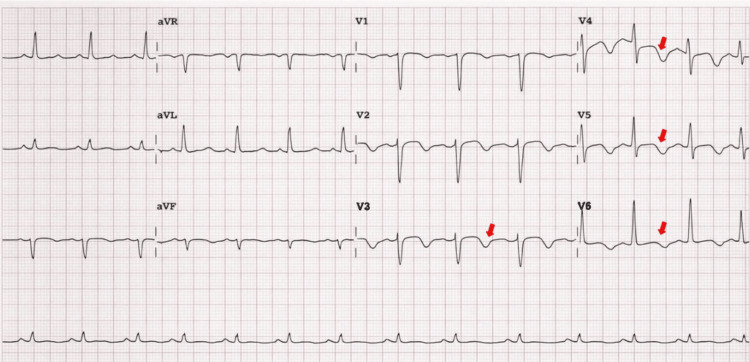
Patient's 12-lead electrocardiogram Shows T-wave inversion in the anterolateral leads, suggestive of myocardial ischemia (red arrows).

The 12-lead electrocardiogram showed T-wave inversion in the anterolateral leads, suggestive of myocardial ischemia (Figure 1).

**Figure 2:**
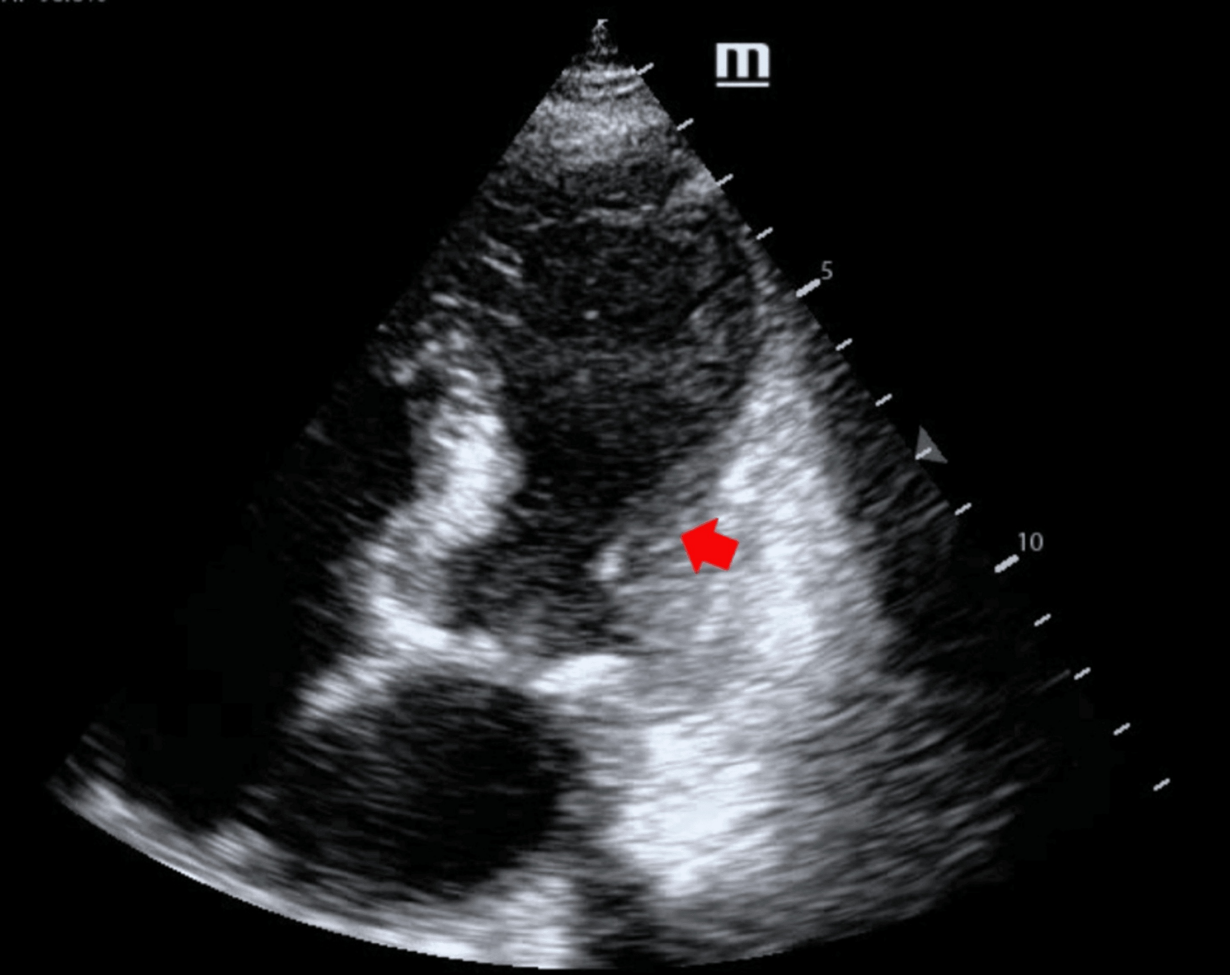
Transthoracic echocardiogram, apical four-chamber view Shows regional abnormalities of left ventricular wall motion, characterized by hypokinesis of the mid-apical segments with preserved basal contraction and hyperdynamic activity, a pattern consistent with stress-induced cardiomyopathy (Takotsubo syndrome) (red arrow).

A bedside transthoracic echocardiogram was performed, revealing regional left ventricular wall motion abnormalities, characterized by hypokinesia of the mid-apical segments associated with basal hyperkinesia. The estimated left ventricular ejection fraction (LVEF) at presentation was approximately 35-40% (Figure 2).

In accordance with the Fourth Universal Definition of Myocardial Infarction [4], the clinical picture was consistent with high-risk non-ST-segment elevation acute coronary syndrome (NSTE-ACS). Risk stratification yielded a Global Registry of Acute Coronary Events Score (GRACE) score [5] of 164 and a Thrombolysis in Myocardial Infarction Score (TIMI) score [6] of five, supporting an early invasive approach. The Interventional Cardiology team was consulted, and the patient was taken to the catheterization laboratory for diagnostic coronary angiography.

Before the procedure, guideline-directed medical therapy was initiated, including loading doses of acetylsalicylic acid (300 mg) and clopidogrel (300 mg), high-intensity atorvastatin (80 mg), anticoagulation with enoxaparin (60 mg), and supportive treatment with omeprazole (40 mg), ondansetron (8 mg), and intravenous buprenorphine (150 mcg) for analgesia.

**Figure 3:**
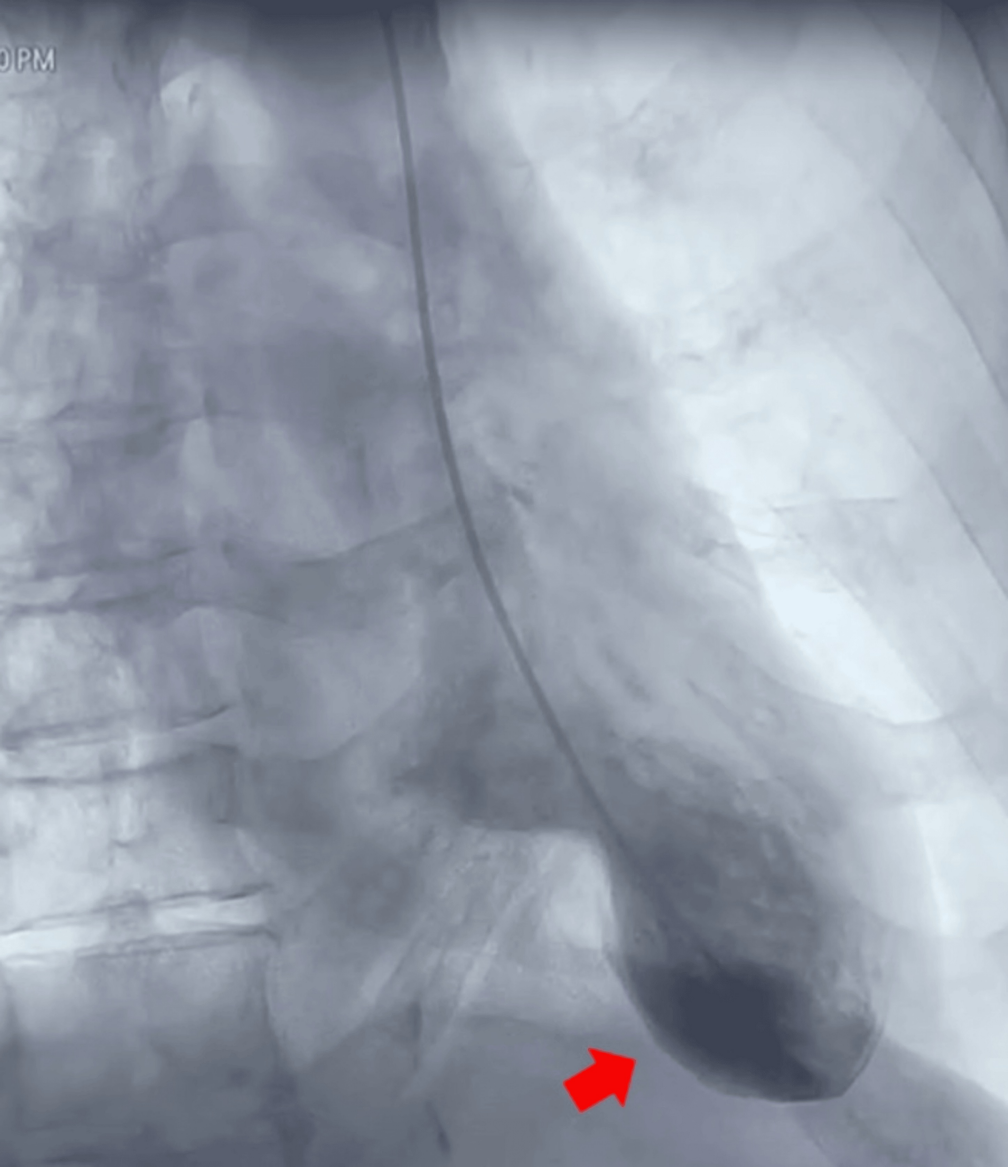
Left ventriculography Demonstrates increased contractility of the basal segments associated with akinesis of the apical segments (inferior, apical, and anterior), configuring the classic pattern of apical bulging, characteristic of Takotsubo syndrome (red arrow).

Angiographic evaluation revealed patent epicardial coronary arteries without flow-limiting stenosis. However, contrast ventriculography demonstrated marked hypercontractility of the basal segments with akinesia involving the apical, inferior, and anterior walls, generating the classic “apical ballooning” configuration (Figure 3).

The absence of obstructive coronary disease in combination with the characteristic ventricular morphology established the diagnosis of Takotsubo syndrome, categorized within the spectrum of myocardial infarction with non-obstructive coronary arteries (MINOCA).

During hospitalization, a targeted assessment for emotional or physical stressors was conducted; however, no clear precipitating event was identified, a scenario reported in up to one-third of cases in the literature. The clinical course was favorable, with no development of heart failure, arrhythmias, or functional deterioration.

The patient showed progressive clinical improvement, remained hemodynamically stable, and experienced no significant functional limitation. She was therefore discharged home with close outpatient follow-up by the Clinical Cardiology service.

A follow-up echocardiogram performed four weeks later showed complete recovery of LVEF (60%) and resolution of regional wall motion abnormalities.

## Discussion

TTS was initially recognized in Japan in 1990 by Sato and colleagues, who described a distinctive pattern of transient ventricular dysfunction resembling a traditional “octopus trap,” or takotsubo, due to the systolic expansion of the apical segments [7]. Since its original description, TTS has been established as a unique form of acute but reversible cardiomyopathy, characterized by temporary impairment of left ventricular systolic performance in the absence of angiographically significant coronary artery obstruction [8]. Several studies have attempted to differentiate acute myocardial infarction from TTS using non-invasive approaches. Recently, an electromechanical mismatch index was proposed based on the correlation between electrocardiographic findings and left ventricular deformation analysis using strain, which could help distinguish stress-induced cardiomyopathy from ST-segment elevation myocardial infarction in the left anterior descending artery territory [9]. 

Epidemiological data indicate that TTS accounts for approximately 1-3% of individuals evaluated for suspected acute coronary syndrome and disproportionately affects postmenopausal women, who comprise nearly 90% of reported cases. In recent years, the recognition of TTS has progressively increased in clinical practice, which may explain the rise in reported cases and the improved understanding of its epidemiological characteristics. Among patients initially managed as ST-segment elevation myocardial infarction, the prevalence of TTS has been reported to range between 5% and 6%. Reports involving Hispanic populations remain comparatively limited in large international registries, despite analyses of national databases having demonstrated that patients of Hispanic origin constitute a significant proportion of hospitalizations for TTS and present distinctive demographic characteristics and clinical outcomes. This underscores the clinical relevance of documenting cases within this demographic context [7,10].

The syndrome is usually triggered by emotional stressors (stress and mental disorders promote cardiovascular disease by activating the hypothalamic-pituitary-adrenal axis and increasing cortisol levels, leading to endothelial dysfunction, inflammation, insulin resistance, and atherosclerosis. Furthermore, chronic activation of the sympathetic nervous system and elevated inflammatory biomarkers contribute to hypertension and the progression of cardiovascular disease [11]) or physical stressors (infections, surgical procedures, intense physical exertion, neurological events, and severe hypoxia). However, in up to one-third of cases, no identifiable triggering factor is found [12], as occurred in our patient, which underscores that the absence of a clear trigger does not rule out the diagnosis. 

The pathophysiology of TTS is multifactorial and not yet fully elucidated. The most widely accepted mechanism involves a catecholamine surge and sympathetic overactivation, leading to calcium overload, oxidative stress, mitochondrial dysfunction, and transient myocardial stunning. Postmenopausal women may be particularly vulnerable due to estrogen deficiency, which contributes to increased sympathetic tone and endothelial dysfunction. Additionally, myocardial edema, microvascular dysfunction, and abnormalities in central autonomic regulation have been described, supporting a heart-brain interaction in the development of TTS [13].

Clinically, TTS presents similarly to acute coronary syndrome, most commonly with chest pain and dyspnea, and less frequently with syncope, arrhythmias, cardiogenic shock, or cardiac arrest [14]. In the present case, severe acute chest pain, ischemic electrocardiographic changes, and elevated cardiac biomarkers initially supported the diagnosis of high-risk NSTE-ACS, warranting invasive coronary evaluation.

Diagnosis is best established using the InterTAK Diagnostic Criteria [7], updated by the International Takotsubo Registry in 2018. These criteria include transient left ventricular dysfunction extending beyond a single coronary territory, possible right ventricular involvement, new electrocardiographic abnormalities, modest troponin elevation with marked natriuretic peptide increase, and exclusion of infectious myocarditis. Importantly, the presence of concomitant coronary artery disease does not rule out TTS. The InterTAK Diagnostic Score further supports clinical suspicion; a score ≥50 strongly favors the diagnosis. Our patient achieved 57 points, reinforcing the diagnosis. Coronary angiography with left ventriculography remains the diagnostic gold standard in the acute setting [15].

Therapeutic strategies in TTS are largely centered on supportive care and must be tailored according to the patient’s hemodynamic profile and the presence of complications. During the acute stage, neurohormonal modulation with angiotensin-converting enzyme inhibitors or angiotensin receptor blockers is commonly employed to promote ventricular functional recovery. Diuretic therapy is appropriate in the setting of volume overload or pulmonary congestion. Although beta-adrenergic blockade may attenuate sympathetic activation and reduce arrhythmic risk in selected cases, its use requires caution in patients with advanced heart failure, hypotension, or marked QT interval prolongation [16].

Beyond the acute phase, continuation of renin-angiotensin system inhibition is generally considered reasonable until normalization of ventricular function is documented. Antiplatelet agents and lipid-lowering therapy are not routinely indicated unless concomitant coronary atherosclerosis is identified. Given the recognized association between emotional stressors and TTS, multidisciplinary follow-up incorporating psychological or psychiatric evaluation may provide additional benefit, particularly in individuals with pre-existing mood disorders [16].

Although TTS is generally considered a reversible condition with a favorable prognosis, up to 20% of patients may develop acute hemodynamic or electrical instability. While women are more frequently affected, men have a higher risk of mortality and major adverse cardiovascular events. Overall mortality has been reported to reach 5.6%, and recurrence occurs in approximately 5% of patients during long-term follow-up [16].

This case presents several noteworthy features that enhance its clinical relevance. First, the patient exhibited a high-risk acute coronary syndrome profile, including markedly elevated cardiac biomarkers and high GRACE and TIMI scores, strongly suggesting an ischemic etiology and warranting an early invasive strategy. Second, no identifiable emotional or physical trigger was found, a scenario reported in a minority of cases, which may further complicate clinical suspicion. Third, the case demonstrates a clear correlation between multimodality imaging findings and clinical evolution, including complete recovery of left ventricular function on follow-up. Together, these elements highlight the diagnostic challenge of TTS and underscore the importance of considering this entity even in patients with high pretest probability for myocardial infarction.

## Conclusions

TTS should be considered in elderly women presenting with acute coronary syndrome-like symptoms, elevated cardiac biomarkers, and ischemic electrocardiographic changes, even in the absence of an identifiable stressor. Transient left ventricular wall motion abnormalities with apical ballooning and non-obstructive coronary arteries are pivotal for diagnosis within the MINOCA spectrum. Recognizing TTS as a part of the MINOCA spectrum is essential to optimize patient care and avoid misdiagnosis.
